# Graphene nanoribbon devices at high bias

**DOI:** 10.1186/s40580-014-0001-y

**Published:** 2014-02-20

**Authors:** Melinda Y Han, Philip Kim

**Affiliations:** 1 Department of Applied Physics and Applied Mathematics, Columbia University, New York, NY 10027 USA; 2 Department of Physics, Columbia University, New York, NY 10027 USA

**Keywords:** 73.23.-b, 85.35.-p

## Abstract

We present the electron transport in graphene nanoribbons (GNRs) at high electric bias conduction. When graphene is patterned into a few tens of nanometer width of a ribbon shape, the carriers are confined to a quasi-one-dimensional (1D) system. Combining with the disorders in the system, this quantum confinement can lead into a transport gap in the energy spectrum of the GNRs. Similar to CNTs, this gap depends on the width of the GNR. In this review, we examine the electronic properties of lithographically fabricated GNRs, focusing on the high bias transport characteristics of GNRs as a function of density tuned by a gate voltage. We investigate the transport behavior of devices biased up to a few volts, a regime more relevant for electronics applications. We find that the high bias transport behavior in this limit can be described by hot electron scattered by the surface phonon emission, leading to a carrier velocity saturation. We also showed an enhanced current saturation effect in the GNRs with an efficient gate coupling. This effect results from the introduction of the charge neutrality point into the channel, and is similar to pinch-off in MOSFET devices. We also observe that heating effects in graphene at high bias are significant.

## Background

The discovery of graphene [1] has enabled intense fundamental and applied research activities in this novel two-dimensional (2D) carbon based electronic system. Electron transport in graphene is substantially different from that of conventional 2D electronic systems owing to the linear energy dispersion relation near the charge neutrality point (Dirac point) in the electronic band structure [2,3]. This unique band structure is fundamentally responsible for the distinct electronic properties of carbon nanotubes (CNTs) [4]. When graphene is patterned into a narrow ribbon, and the carriers are confined to a quasi-one-dimensional (1D) system, we expect the opening of an energy gap. Earlier theoretical work showed that this energy gap depends on the width and crystallographic orientation of the graphene nanoribbon (GNR) [5–7], similar to CNTs. The first experimental work on GNRs [8–10] has shown that a transport gap can indeed be open up by patterning graphene into nanometer size ribbons or constrictions. The resulting transport gap formation can be most simply attributed to quasi-onedimensional (1D) confinement of the carriers, which induces an energy gap in the single particle spectrum [5–7,11–13]. Detailed experimental studies of disordered graphene nanoribbons (GNRs) [14–20], however, suggest that this observed transport gap may not be a simple band gap. In an effort to explain these experimental results, various theoretical explanations for the transport gap formation in disordered graphene nanostructures have been proposed, including models based on Coulomb blockade in a series of quantum dots [21], Anderson localization due to edge disorder [22–24], and a percolation driven metal-insulator transition [25]. In order to distinguish between these different scenarios, systematic experiment including treatment of both disorder induced localization and electron-electron interaction is required.

In our recent experiment [20], we carried out systematic studies of the scaling of the transport gap in GNRs of various dimensions. From this scaling of several characteristic energies with GNR width (*W*) and length (*L*), we find evidence of a transport mechanism in disordered GNRs based on hopping through localized states whose size is close to the GNR width. We found that At the charge neutrality point, a length-independent transport gap forms whose size is inversely proportional to the GNR width. In particular, we found that in this gap, electrons are localized, and charge transport exhibits a transition between thermally activated behavior at higher temperatures and variable range hopping at lower temperatures. By varying the geometric capacitance, we find that charging effects constitute a significant portion of the activation energy.

Extending this earlier work, in this review, we examine the electronic properties of lithographically fabricated GNRs with widths in the tens of nanometers. Here we investigate the transport behavior of devices biased up to a few volts, a regime more relevant for electronics applications. We will first address characteristics of graphene at high bias which are not specific to graphene nanoribbons, then we address GNRs at high bias specifically. Graphene devices operated at high source-drain bias show a saturating *I*−*V* characteristic. This decrease in conductivity at high applied electric field is described by carrier velocity saturation due to optical phonon emission. This result is analogous to the high bias results obtained CNTs. In a well known experiment, Yao *et. al.* [26] found that current in metallic single wall carbon nanotubes saturates at high electric field. Their result is explained in terms of zone-boundary optical phonon emission from high energy electrons. At high electric fields, a steady-state population is developed between right and left moving charge carriers with a maximum energy difference corresponding to the phonon energy $\hbar \Omega =160$ meV, leading to a saturated current of $(4e/h)/(\hbar \Omega)\approx 25~\mu \mathrm {A}$. A slightly different behavior was reported in semiconducting single wall carbon nanotubes by Chen and Fuhrer [27]. In these devices, current does not saturate completely, and the transport is described by an electric field dependent carrier velocity. The authors fit their data with a model based on a carrier velocity that saturates to a constant value at high electric field and a carrier density dependent on the local potential along the device. They find a saturation velocity of 2 × 10^7^ cm/s in their device. These results demonstrate the feasibility of 1D GNR devices for electronic applications with a proper bandgap engineering.

## Graphene nanoribbon fabrication

**Figure 1:**
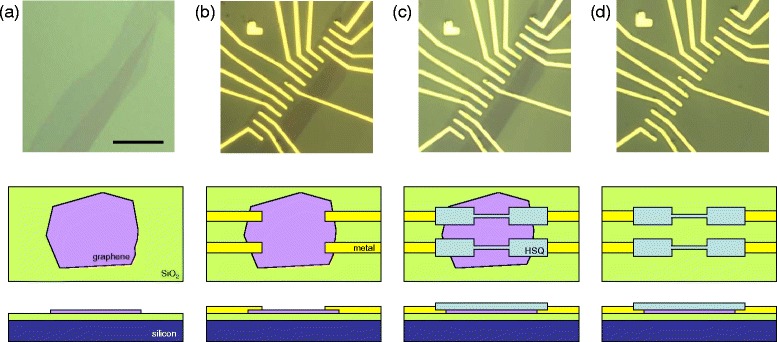
**Process flow for graphene nanoribbon fabrication.** Optical microscope image (top row), cartoon top view (middle row), and cartoon side view (bottom row), for each of four major processing steps. **(a)** Graphene deposition on Si/SiO_2_ substrate. **(b)** E-beam lithography fabrication of metal electrodes. **(c)** Patterning of negative e-beam resist etch mask. **(d)** Removal of unprotected graphene by oxygen plasma etching. Scale bar in optical image is 20 μm, all four optical images have the same scale.

**Figure 2:**
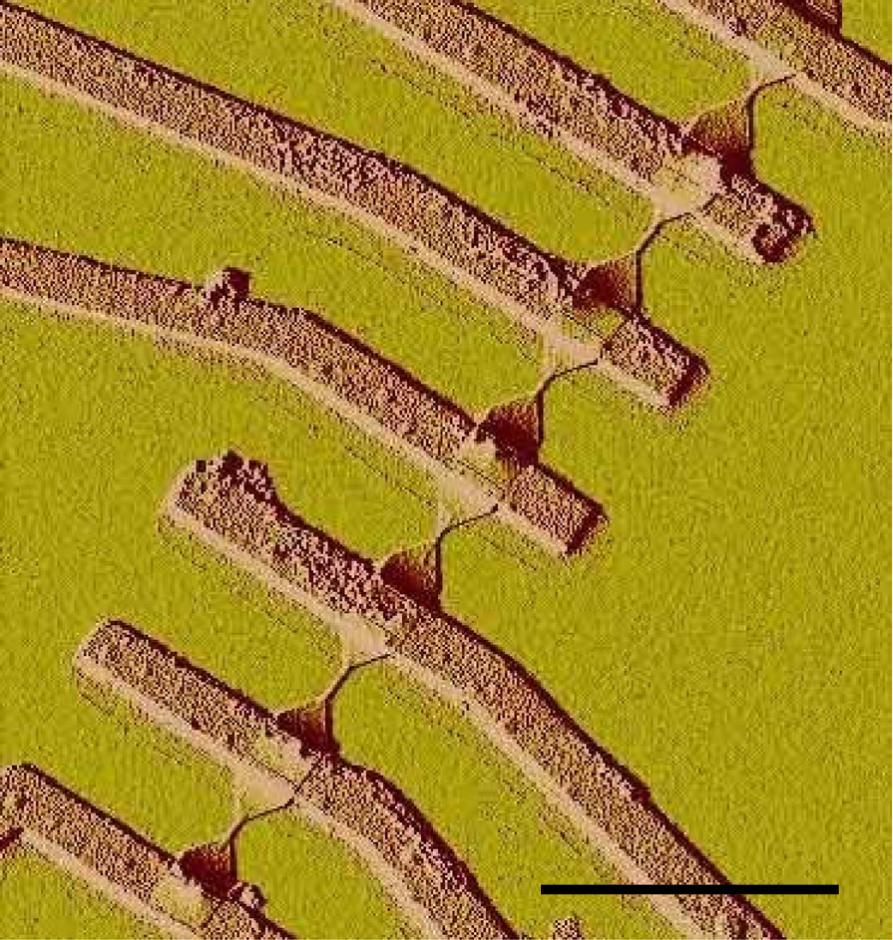
**Atomic force microscope (AFM) image of the device in Figure** 1 **.** The ribbons in this image correspond to the lower six ribbons in the optical image in Figure 1(d). Scale bar is 4 μm.

GNRs used in this study were fabricated by lithgrapically patterned structure from mechanically exfoliated graphene. The process flow is outlined in Figure 1. Briefly, we begin with exfoliated graphene, fabricate metal electrodes using standard electron beam (e-beam) lithography procedures, pattern an etch mask using an negative e-beam resist, and etch away unprotected graphene using an oxygen plasma etch. An atomic force microscope (AFM) image of a finished device is shown in Figure 2.

Once a suitable piece of graphene has been deposited and identified using the procedure described above, the next step is to electrically contact the graphene with metal electrodes using e-beam lithography. We begin by spinning on a layer of poly(methyl methacrylate) (PMMA) e-beam resist and baking on a hotplate at 180°C for 2 minutes. Then we use e-beam lithography to write a 2 mm by 2 mm grid of alignment marks at roughly the location of the graphene, and develop in a solution of methyl isobutyl ketone:isopropal alcohol (MIBK:IPA) 1:3 for 5–10 seconds. This quick development leaves alignment mark “holes” in the PMMA, which we use for alignment in the following e-beam lithography step, eliminating the need for metal alignment mark deposition or another PMMA spin step. Electrodes are patterned in this PMMA layer with e-beam lithography, using an optical image of the sample with the alignment mark holes for design and alignment. Thermal evaporation is then used to deposit 1–2 nm of chrome and 25–50 nm of gold, and the chip is placed in acetone overnight at room temperature for lift-off (Figure 1(b)).

Once the graphene has been successfully contacted with Cr/Au electrodes, we create an etch mask to define the nanoribbons. A negative tone e-beam resist, hydrogen silsesquioxane (HSQ) (2% in MIBK) is spun on to the chip (at 4000 rpm, for a typical film thickness of 14 nm). We use HSQ as the resist for this step because a negative resist is ideal for creating a small etch mask, and because with HSQ we can obtain small feature sizes. The etch mask is written at a relatively high e-beam dose (1300 μC/c*m* ^2^ for the ribbons in our 30 keV system, with lower doses for larger features) and developed in a solution of 0.26N tetramethylammonium hydroxide (TMAH) in water for 1 minute (Figure 1(c)).

After defining the etch mask, the graphene is ready to be etched. The device is exposed to oxygen plasma in a Technics reactive ion etcher (RIE) with 200 mTorr *O*_2_ at 50 W for 5–10 seconds. These conditions etch graphene at a rate of about one layer per second, so that unprotected single layer and few-layer graphene are etched away cleanly (Figure 1(d)). The finished device (Figure 2) is then ready to be wirebonded and measured.

The devices measured in this experiment are back-gated and dual-gated etched graphene devices. Graphene devices often fail or change drastically and irreversibly when the current density per unit width exceeds a threshold of ∼2 mA/μm. We operate the device at currents below this threshold. Current-voltage characteristics at varying gate voltages were measured for 17 ribbon devices with a range of widths and lengths, and three “wide” devices with *W* = 200 nm, in order to compare to the behavior of non-ribbon devices.

## Saturating behavior fits a velocity saturation model

**Figure 3:**
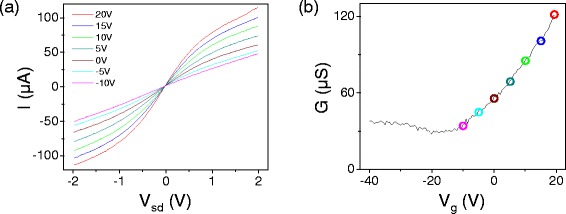
**High bias transport data from a ribbon with W=70 nm and L=500 nm measured at room temperature in vacuum.** **(a)** Current vs. source drain bias at varying gate voltages *V*_*g*_, as shown in the legend. **(b)** Conductance vs. gate voltage for the same device at a source drain bias of *V*_*sd*_ = 200 meV.

**Figure 4:**
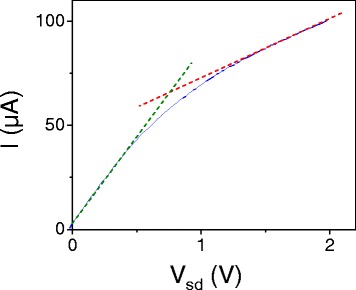
**Current-voltage characteristic for the curve with** ***V***_***g***_ **−** ***V***_***CNP***_ **= -30V shown in Figure** 3 **(a).** The distinct slopes at low and high *V*_*sd*_ resulting from the saturation behavior are highlighted with green and red dashed lines, respectively.

Figure 3 shows a plot of current vs source-drain bias for varying gate voltages in a back-gated device. We focus here on the curves taken at densities far from the charge neutrality point, such as the curve singled out in Figure 4. Here we see that at low bias the slope of the curve is constant, and at high bias the curve turns down, approaching a linear behavior with a reduced slope.

To describe this saturating decrease in conductivity, we propose a model based on an electric field-dependent carrier velocity *v*_*d*_(*E*) of the form:

(1) $$ v_{d}(E)=\left(\frac{1}{\mu_0E}+\frac{1}{v_{sat}} \right)^{-1}  $$

where *μ*_0_ is the low field mobility and *v*_*sat*_ is a phenomenologically introduced saturation velocity. The total current through the device is given by

(2) $$ I=jW=-{nev}_dW  $$

We assume that the capacitance to the back gate dominates in determining the charge density in the channel, so that

(3) $$ {ne}=C_{g}\left(V(x)-(V_{g}-V_{CNP})\right)=C_{g}(V-V_{0})  $$

where *V* = *V*(*x*) is the potential at position x along the channel, and we have defined *V*_0_≡*V*_*g*_−*V*_*CNP*_. Using the relation *E*=*d* *V*/*d* *x*, we have

(4) $$ I^{-1}=-\frac{1}{{WC}_{g}(V-V_{0})}\left(\frac{1}{\mu_{0}dV/dx}+\frac{1}{v_{sat}}\right)  $$

Rearranging terms and integrating gives the current

(5) $$ I={WC}_{g}(V_{0}-V_{b}/2)\frac{\mu_{0} V_{b}/L}{1+\mu_{0} V_{b}/v_{sat}L}  $$

In its limiting forms, Equation 5 for the current qualitatively gives the behavior seen in Figure 4. At low *V*_*b*_, current is linear in *V*_*b*_ with a conductivity *WC*_*g*_ *V*_0_ *μ*_0_/*L*, determined by the low field mobility, as expected. At high *V*_*b*_, current is again linear in *V*_*b*_, but now with a conductivity of *WC*_*g*_ *v*_*sat*_/2 and an offset determined by the gate voltage. At low fields, the variation in carrier density is small and the linear *I–V* results from the linear form of *v*_*d*_(*E*)≈*μ*_0_ *E* in this regime. At high fields, *v*_*d*_ approaches a constant value *v*_*sat*_, and the linear dependence of the carrier concentration on *V*_*b*_ is responsible for an *I–V* characteristic approaching linear behavior. Note this is in contrast to the case of carbon nanotubes, where there are a set number of conducting channels, so that the current saturates with the drift velocity.

**Figure 5:**
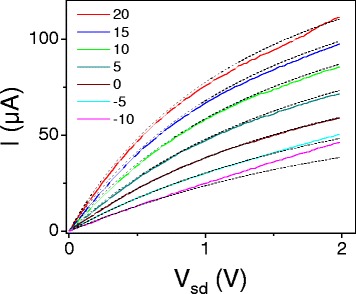
**Fit of the current model in Equation** 5 **to the data in Figure** 3 **.** The legend gives the gate voltage for each sweep, dashed lines are fits to each sweep.

The expression in Equation 5 for *I*=*I*(*V*_*b*_) was fit to the *I–V* characteristics in Figure 3; the result is shown in Figure 5. For ribbon devices, the geometry is not well approximated by a parallel plate capacitor, so the gate capacitance was calculated numerically.For the device in Figure 5, the capacitance was calculated to be 47.5 nF/cm^2^ using a numerical calculation based on the finite element method. The model fits well for curves taken at densities far at high carrier densities, and begins to break down for curves measured near the charge neutrality point, as seen in Figure 5 for *V*_*g*_=−10 V. This fit has two free parameters, *v*_*sat*_ and *μ*_0_. For this dataset, this model gives *μ*_0_ values between 400 and 600 cm^2^/Vs, compared to the value of 700 cm^2^/Vs from low bias sweeps of *G–V*_*g*_.

**Figure 6:**
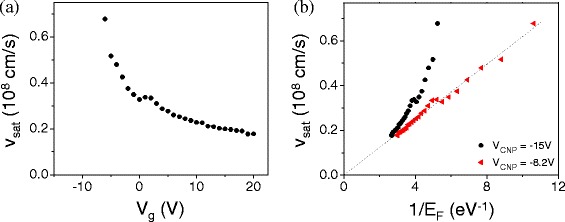
**Saturation velocity values extracted from the fits in Figure** 5 **.** **(a)** Saturation velocity *v*_*sat*_ as a function of the gate voltage *V*_*g*_. **(b)** *v*_*sat*_ vs the inverse of the Fermi energy *E*_*F*_. Black circles and red triangles correspond to a conversion of *V*_*g*_ to *E*_*F*_ using *V*_*CNP*_=−15 V and *V*_*CNP*_=−8 V, respectively. Dashed line is a linear fit to this data.

The values of *v*_*sat*_ obtained from this fit are plotted against *V*_*g*_ in Figure 6(a). In Figure 6(b), we plot *v*_*sat*_ against the inverse of the Fermi energy

(6) $$ E_{F}=\hbar v_{F} \sqrt{\pi C_{g}(V_{g}-V_{CNP})}  $$

Converting *V*_*g*_ to *E*_*F*_ involves the value of *V*_*CNP*_, which commonly drifts throughout measurement due to changes in adsorbed molecules and positions of trapped charges. Here the black circles correspond to conversion of *V*_*g*_ to *E*_*F*_ using *V*_*CNP*_ = −15 V, the same value used in Equation 5 for the original fit. Red triangles represent a conversion to *E*_*F*_ using *V*_*CNP*_ = −8 V so that the a linear fit of *v*_*sat*_ vs. $E_{F}^{-1}$ intersects the origin.

In order to understand the inverse relationship between *v*_*sat*_ and *E*_*F*_, we seek a physical understanding of the electric field dependent carrier velocity, or drift velocity, in Equation 1. This expression corresponds to scattering by optical phonons, which would produce an electric field dependent mean free path. By Matthiessen’s rule, mean free paths add as

(7) $$ \frac{1}{l}= \frac{1}{l_{sc}}+\frac{1}{l_{op}}  $$

where *l* is the total mean free path and *l*_*sc*_ is the mean free path for elastic impurity scattering and quasi-elastic acoustic scattering, and *l*_*op*_ is the mean free path for optical phonon emission. If electrons are immediately scattered upon reaching the optical phonon energy, so that

(8) $$ l_{op}=\frac{\hbar\Omega}{eE}  $$

where *E* is the electric field and *Ω* is the relevant optical phonon frequency, then the mobility *μ* is given by

(9) $$ \frac{1}{\mu} = \frac{1}{\mu_{0}}+\frac{E}{v_{sat}}  $$

This form of the mobility results in the expression for the drift velocity *v*_*d*_ = *μ* *E* given in Equation 1. For electrons and holes in graphene, which have a constant carrier velocity of *v*_*F*_, drift velocity can be understood as the time averaged velocity of carriers when scattering is taken into account.

From the above calculation we see that our phenomenological velocity saturation model can be understood in terms of a picture where electrons scatter by optical phonon emission upon reaching the phonon energy $\hbar \Omega $ under the influence of the applied electric field. With this in mind, we derive an expression for current density using a different approach, in order to gain insight into our measured values for the saturation velocity. Current density is given by

(10) $$ \vec{j}=-e\int d\vec{k} D_{k} \vec{v}(\vec{k}) g(\vec{k})  $$

where *D*_*k*_ = 2/(2*π*)^2^ is the density of electronic states in k-space, $\vec {v}(\vec {k})=v_{F}$ is the electron velocity, and $g(\vec {k})$ is the distribution function. In the relaxation time approximation, we have

(11) $$ g(\vec{k})=g^{0}(\vec{k})-e\vec{E}\cdot \vec{v}(\vec{k}) \tau(\epsilon(\vec{k}))\left(-\frac{\partial f}{\partial \epsilon} \right)  $$

where $g^{0}(\vec {k})$ is the equilibrium distribution function, *τ* is the relaxation time, and *f* is the Fermi-Dirac distribution function. For a device with its length in the *x* direction, we seek $\vec {j}=j\hat {x}$, so we consider only $\vec {E}=E\hat {x}$, and

(12) $$ \vec{E}\cdot \vec{v}(\vec{k})={Ev}_{F}\cos{\theta}  $$

where *θ* is the angle between $d\vec {k}$ and $\vec {E}$. We assume that electrons are immediately scattered upon reaching the energy threshold for phonon emission, giving

(13) $$ \tau=\frac{\hbar\Omega}{{eEv}_{F}}  $$

So that for Equation 10 we have

(14) $$ j=e\int\frac{dk}{\pi^{2}}v_{F}\cos^{2}\theta\hbar\Omega\left(-\frac{\partial{f}}{\partial{\epsilon}}\right)|_{\epsilon=\hbar v_{F} k}  $$

In polar coordinates

(15) $$ \begin{aligned} j&=e\int_{0}^{\infty}\frac{dk}{\pi^{2}}\int_{0}^{2\pi}d\theta v_{F}\cos^{2}\theta\hbar\Omega\delta(\hbar v_{F}k-E_{F})\\ &=\frac{e}{\pi}\Omega\frac{E_{F}}{\hbar v_{F}} \end{aligned}  $$

At high fields, we assume *j*=*nev*_*sat*_ and use $E_{F}=\hbar v_{F}\sqrt {\pi n}$ to obtain

(16) $$ \frac{v_{sat}}{v_{F}}=\frac{\hbar\Omega}{E_{F}}  $$

Using this expression with *v*_*F*_=10^8^ cm/s [2,3], we obtain a value of ${\hbar \Omega =62.0}$ meV from the linear fit (dashed line) in Figure 6(b). This is well below the value of the longitudinal zone-boundary phonon for graphene, which has ${\hbar \Omega =200}$ meV [28]. We suggest that our measured phonon energy corresponds to the SiO_2_ surface phonon energy ${\hbar \Omega =55}$ meV [29–31], although we note that values measured in other ribbon devices of different geometries vary widely (from ≈22 meV to ≈120 meV), possibly due to discrepancies in determining the relevant device geometry, the corresponding capacitance, and the position of the charge neutrality point.

## Top-gated graphene devices show an enhanced current saturation effect

**Figure 7:**
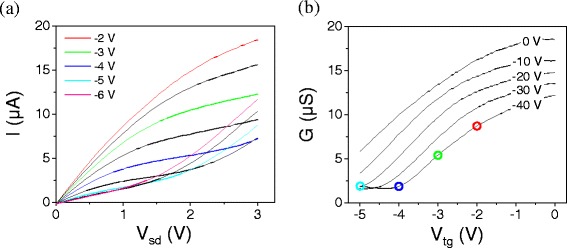
**Current-voltage characteristics for a dual-gated device with W=35 nm and L=2 μm measured at room temperature in vacuum.** **(a)** *I–V*_*sd*_ at constant *V*_*bg*_= -40 V and varying *V*_*tg*_. Select curves are shown in color and have *V*_*tg*_ values as noted in the legend, the black curves fall between these curves at 0.5 V increments. **(b)** *G-V*_*tg*_ at varying *V*_*bg*_ values, as noted on each curve, measured with *V*_*sd*_=1 meV. Colored dots correspond to the gate voltage positions where the colored curves in Figure 7 (a) were measured.

**Figure 8:**
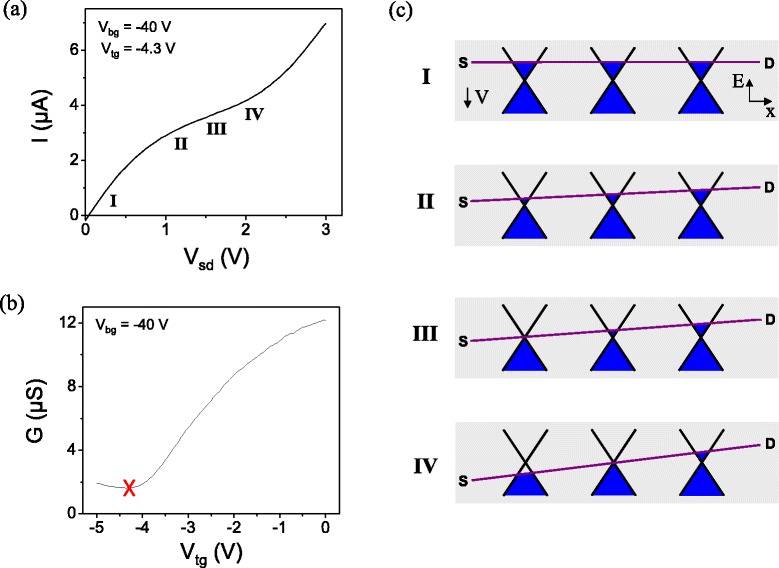
**Kink and current saturation.** **(a)** An *I–V*_*sd*_ curve for the device in Figure 7, highlighting the current saturation “kink” behavior. **(b)** *G-V*_*tg*_ for the same device; the red “x” highlights the low bias (*V*_*sd*_=1 meV) conditions corresponding to the curve in (a). **(c)** Cartoon schematic of the Fermi level in the channel for each condition (I–IV) marked in (a). Dirac cones with different Fermi levels along the length of the channel result from carrier density variation along the channel under the influence of a strong *V*_*sd*_.

In dual-gated devices, we observe a velocity saturation behavior similar to that the back-gated device behavior described above. However, we also see an enhanced current saturation at certain gate voltage combinations, as first reported in Reference [32]. Figure 7 shows current-voltage characteristics and corresponding conductance-gate voltage sweeps for a dual-gated device with *W*=35 nm and *L*=2 μm. At combinations of *V*_*bg*_ and *V*_*tg*_ near the charge neutrality point, we see a “kink” in the *I–V* curve, where the current first begins to flatten out, then turns upwards again. Figure 8 highlights this behavior in one *I–V* curve from the same device. This effect is specific to top-gated devices, where the strong capacitive coupling allows the bias voltage to dominate the carrier density in the channel.

The “kink” effect in graphene is similar to pinch-off in traditional MOSFETs, where a strong bias voltage pulls the quasi-Fermi level at one end of the channel into the charge-depleted bandgap. In graphene, where there is no bandgap, this results in a transition within the channel from one carrier type (electrons or holes) to the other. In a device that is n-type, as in Figure 8(c)(I), a positive source-drain voltage (applied to the source) depletes the electron density in the channel near the source (II). At sufficiently strong positive bias voltage, the bias voltage begins to pull holes into the channel, so a region of the channel is at charge neutrality and contributes a large resistance (III). As bias is further increased, hole density at the source also increases, so conductivity increases again (IV). In Reference [32] we showed that wide plateaus in current could be achieved when this “kink” effect is made to coincide with velocity saturation.

## Heating effects can overcome transport gap at high bias

The results discussed above come from graphene nanoribbons measured at high bias, but the key features of the data, saturation velocity at strong electric fields and the “kink” effect in the current for top-gated devices, are also seen in wide graphene devices [32]. This leads to the question, how are nanoribbons different from wide, non-ribbon devices when operated at high bias? Here we present the preliminary results of a comparison between dual-gated ribbons and wide devices and so far find no major differences in their performance. This result is only preliminary because the widths of the GNRs in this experiment are not well specified within the range of *W*≈ 20–60 nm. The widths of nanoribbons lying underneath the dielectric and metal layers cannot be accurately measured in this device geometry. Estimates of the width can be made based on the expected width dependence of the low-temperature transport characteristics *Δ* *m* and *Δ* *V*_*b*_ from the analysis in earlier work [20]. From these comparisons, it is estimated that the ribbons used in this experiment have *W*≈50 nm. Ribbons of this width are narrow enough to behave distinctly from “wide” ($W \gtrsim 100$ nm) devices at low temperatures and low bias, but as we shall see below, they may not be narrow enough show a difference in transport characteristics at high bias. Ribbons as narrow as *W*≈ 15–20 nm are achievable by our fabrication methods, so measurements of narrower devices with larger transport gaps may still reveal distinct device behavior.

In comparing gapped graphene nanoribbons to wide graphene with no gap, there are several differences we may expect to see. First, since graphene nanoribbons have a strongly suppressed current at energies inside the gap, we may see an increased transconductance. Also, we could see larger and more fully saturated current in the “kink” region, as the presence of a gap causes the “kink” to more closely resemble pinch-off in a traditional MOSFET. We may also see the effects of edge roughness. In narrow ribbons where edge roughness constitutes a significant portion of the total ribbon width, this could lead to a decrease in maximum current carrying capabilities, or cause the devices to degrade more quickly.

**Figure 9:**
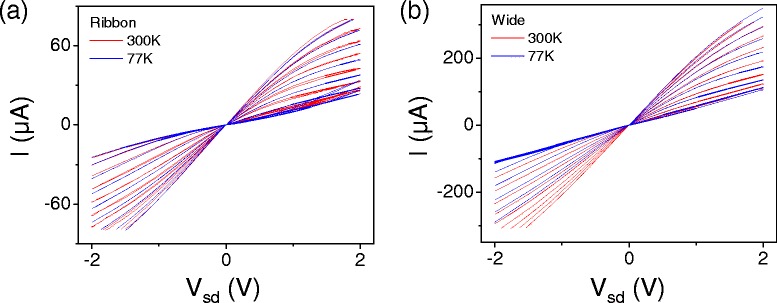
**Top gated transfer characteristic for nanoribbons.** *I–V*_*sd*_ characteristics at constant *V*_*bg*_=0 with *V*_*tg*_ varying from 0 to -8 V, measured at T=77 K and T=300 K for **(a)** a ribbon device (*W*≈50 nm) and **(b)** a wide graphene device (*W*=200 nm). Both devices have *L*=500 nm.

Figure 9 shows *I–V* characteristics for graphene devices taken at two different temperatures, 77 K and 300 K. The *I–V* curves do not change significantly between the two temperatures. Since we expect to see thermal effects in the conductivity even away from the charge neutrality point, this suggests that the effective temperature in the device is similar at both 77 K and 300 K, in other words, other heating in the system dominates over the ambient temperature up to 300 K.

**Figure 10:**
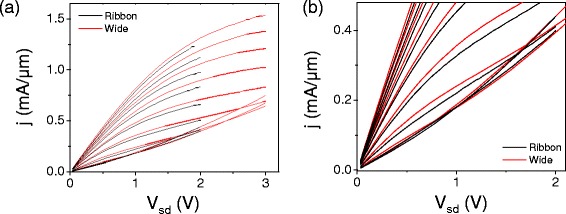
**Current density versus bias voltage at fixed gates.** **(a)** Current density *j*=*I*/*W* at high *V*_*sd*_ at constant *V*_*bg*_ and varying *V*_*tg*_ for the two devices in Figure 9. **(b)** Same data as in (a), enlarged to show behavior near in the region of the “kink”.

As a straightforward method to directly compare ribbon devices with wide devices, we compare the scaled current density per width *j*=*I*/*W* for two devices, a ribbon device with width *W*≈50 nm and a wide device with *W*=200 nm, both with length *L*=500 nm, shown in Figure 10. Here we can see that in the ribbon device, there is no difference in the size or shape of the kink behavior (Figure 10(b)), and only a minor difference in transconductance. From this data we see that a ∼50 nm wide graphene nanoribbon shows no major differences in behavior from a wide device when operated at high bias.

To understand the similarity in behavior between 50 nm and 200 nm wide devices, we compare the gap size of the ribbon device with the relevant thermal effects in the system; if the available thermal energy in the system is larger than the gap, the effect of the gap will be washed out by thermally activated charge carriers. In earlier work [20], we found that there are three different ways to measure the size of the gap: *Δ*_*m*_, from the gate voltage, *Δ* *V*_*b*_, from the bias voltage, and *E*_*a*_, from the activation energy for nearest neighbor hopping. Here we are concerned with current flow at high bias, so *Δ* *V*_*b*_ is the most relevant of these scales for distinguishing the on and off states of the device, though *E*_*a*_ will determine the leakage current in the off state. For the 500 nm long devices studied here, these values are similar. Since *Δ* *V*_*b*_ has a strong length dependence, if we wish to increase *Δ* *V*_*b*_ we can increase the device length *L*, with the trade-off of an increased the resistance and therefore a decreased current.

**Figure 11:**
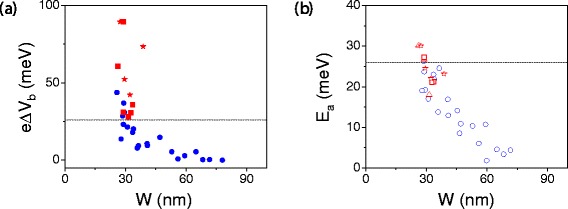
**Bias gap and activation energy versus GNR width.** **(a)** Measures of bias gap vs width from the data in reference [20]. **(b)** The activation erengy estimated from Arrhenius relation. The room temperature thermal energy 26 meV is highlighted with the horizontal dashed lines.

We consider two heating effects in this experiment. First, we compare the size of the gap with the thermal energy at room temperature, *k*_*B*_ *T*≈26 meV. Figure 11 shows how the relevant gap sizes compare with the room temperature thermal energy. We see that *Δ* *V*_*b*_ can be easily made greater than 26 meV by decreasing the ribbon width to below 30 nm or increasing ribbon length. However, only the narrowest ribbons shown here have a large enough *E*_*a*_; narrower ribbons would be needed to ensure a low thermally activated leakage current.

If heating effects raise the device temperature above room temperature, then heating effects will be more relevant than ambient temperature effects. Several recent works [33–35] address the topic of heating in graphene at high bias. From the ribbon device data in Figure 9(a), we can expect to see dissipated electrical power *P*=*I* *V* of up to ≈350 kW/cm^2^, though power dissipation may be lower in the optimal operating regime for device applications. From the results in Reference [35] for temperature vs. power per area, this power dissipation corresponds to a temperature of 1350 K, or a thermal energy of 116 meV. From this it is clear that the thermal energy from heating greatly exceeds that from the ambient temperature, but this result was from a back-gated device. In a dual-gated device geometry, the top-gate dielectric and electrode may act as a heat sink and decrease the effect of heating.

**Figure 12:**
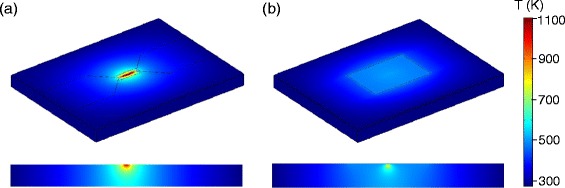
**Thermal modeling of GNRs at high bias.** Thermal modeling of **(a)** back-gated and **(b)** dual-gated graphene nanoribbon devices. Top row is a full 3D view of each device (on a 3 *μ*m by 4 *μ*m rectangle), bottom row is a cross-sectional slice taken midway across the ribbon (showing the oxide thickness of 285 nm), colorbar is the same for all plots.

In Figure 12, we model the heat sinking effects of a gate dielectric and top gate on a hot ribbon. This was done in the COMSOL Multiphysics finite element modeling package by assigning a heat flux to the ribbon such that maximum temperature in a back-gated device is ∼1100 K, shown in Figure 12(a). The graphene ribbon and graphene leads were assigned a thermal conductivity of 5000 K [36] and a thickness of 3.4; heat dissipation was also allowed through the 285 nm SiO_2_ layer to the Si substrate below. In Figure 12(b), a 30 nm SiO_2_ gate dielectric and a 30 nm gold top gate are added to the same model, again allowing heat dissipation into the gate dielectric and the top gate. Here, SiO_2_ was used in place of HSQ because they are expected to have similar material properties. In this model, the maximum temperature is decreased to 825 K. If the top gate thickness is increased to 100 nm to allow for more heat sinking, the temperature decreases only slightly more, to 812 K.

The gate dielectric actually used in the experiment consists of HSQ/ HfO_2_ with thicknesses of 15/15 nm. Hafnium dioxide has a much higher thermal conductivity than silicon dioxide (23 W/m·K for HfO_2_ versus 1.4 W/m·K for SiO_2_). When the model is changed to include the proper layer thicknesses of each dielectric, the maximum nanoribbon temperature decreases to 680 K, which corresponds to an energy of 59 meV. This is the behavior we can expect to see in the actual device measured in Figures 9 and 10. From Figure 11 it is clear that *E*_*a*_ and *Δ* *V*_*b*_ are both far below this energy, so thermally activated carriers easily wash away any gap-related effects we might have seen in the transport at high bias.

Heat sinking could be greatly improved by removing the HSQ, such as by an hydrofluoric acid etch, and depositing ≈15 nm of hafnia only as the dielectric. In this geometry, the dielectric would be thinner and more thermally conductive, allowing for more efficient heat dissipation to the metal top gate. For the same heating conditions, this device construction would result in a maximum nanoribbon temperature of 460 K. The corresponding energy, 40 meV, is a gap size easily achievable by our nanoribbon fabrication methods. We note that from the results in our earlier work [20], the addition of a top gate tends to decrease *E*_*a*_, but as the top-gated geometry provides very good heat sinking, and top gates will ultimately be needed for optimized device design, we see this as the best route for development of a graphene nanoribbon device that retains its gapped behavior at high bias.

## Conclusions

In this review we have described a saturating *I–V* characteristic in graphene devices operated at high source-drain bias, and described the behavior using a model where surface phonon emission results in a carrier velocity that saturates to a Fermi energy dependent value at high applied electric field. We showed that for top-gated graphene devices have an enhanced current saturation effect at certain gate voltage combinations. This effect results from the introduction of the charge neutrality point into the channel, and is similar to pinch-off in MOSFET devices. We observe that heating effects in graphene at high bias are significant, and very narrow ribbons with a strongly heat sinking device design are required to produce a device where confinement-induced gap effects dominate over the effects of heating.

## References

[1] Novoselov KS, Geim AK, Morozov SV, Jiang D, Zhang Y, Dubonos SV, Grigorieva IV, Firsov AA (2004). Electric field effect in atomically thin carbon films. Science.

[2] Novoselov KS, Geim AK, Morozov SV, Jiang D, Katsnelson MI, Grigorieva IV, Dubonos SV, Firsov AA (2005). Two-dimensional gas of massless dirac fermions in graphene. Nature.

[3] Zhang Y, Tan Y-W, Stormer HL, Kim P (2005). Experimental observation of the quantum hall effect and berry’s phase in graphene. Nature.

[4] Dresselhaus MS, Dresselhaus G, Saito R (1995). Physics of carbon nanotubes. Carbon.

[5] Nakada K, Fujita M, Dresselhaus G, Dresselhaus MS (1996). Edge state in graphene ribbons: Nanometer size effect and edge shape dependence. Phys. Rev. B.

[6] Wakabayashi K, Fujita M, Ajiki H, Sigrist M (1999). Electronic and magnetic properties of nanographite ribbons. Phys. Rev.

[7] Son Y, Cohen ML, Louie SG (2006). Energy gaps in graphene nanoribbons. Phys. Rev. Lett.

[8] Han MY, Özyilmaz B, Zhang Y, Kim P (2007). Energy band-gap engineering of graphene nanoribbons. Phys. Rev. Lett.

[9] Chen Z, Lin Y-M, Rooks MJ, Avouris P (2007). Graphene nano-ribbon electronics. Physica. E.

[10] Li X, Wang X, Zhang L, Lee S, Dai H (2008). Chemically derived, ultrasmooth graphene nanoribbon semiconductors. Science.

[11] Ezawa M (2006). Peculiar width dependence of the electronic properties of carbon nanoribbons. Phys. Rev.

[12] Brey L, Fertig HA (2006). Electronic states of graphene nanoribbons studied with the dirac equation. Phys. Rev. B.

[13] Barone V, Hod O, Scuseria GE (2006). Electronic structure and stability of semiconducting graphene nanoribbons. Nano Lett.

[14] Ponomarenko LA, Schedin F, Katsnelson MI, Yang R, Hill EW, Novoselov KS, Geim AK (2008). Chaotic dirac billiard in graphene quantum dots. Science.

[15] Stampfer C, Güttinger J, Hellmüller S, Molitor F, Ensslin K, Ihn T (2009). Energy gaps in etched graphene nanoribbons. Phys. Rev. Lett.

[16] Molitor F, Jacobsen A, Stampfer C, Güttinger J, Ihn T, Ensslin K (2009). Transport gap in side-gated graphene constrictions. Phys. Rev. B.

[17] Todd K, Chou H, Amasha S, Goldhaber-Gordon D (2009). Quantum dot behavior in graphene nanoconstrictions. Nano Lett.

[18] Liu X, Oostinga JB, Morpurgo AF, Vandersypen LMK (2009). Electrostatic confinement of electrons in graphene nanoribbons. Phys. Rev. B.

[19] Gallagher KTP, Goldhaber-Gordon D (2010). Disorder-induced gap bahavior in graphene nanoribbons. Phys. Rev. B.

[20] Han MY, Brant JC, Kim P (2010). Electron transport in disordered graphene nanoribbons. Phys. Rev. Lett.

[21] Sols F, Guinea F, Neto AHC (2007). Coulomb blockade in graphene nanoribbons. Phys. Rev. Lett.

[22] Gunlycke D, Areshkin DA, White CT (2007). Semiconducting graphene nanostrips with edge disorder. Appl. Phys. Lett.

[23] Evaldsson M, Zozoulenko IV, Xu H, Heinzel T (2008). Edge-disorder-induced anderson localization and conduction gap in graphene nanoribbons. Phys. Rev. B.

[24] Querlioz D, Apertet Y, Valentin A, Huet K, Bournel A, Retailleau SG, Dollfus P (2008). Suppression of the orientation effects on bandgap in graphene nanoribbons in the presence of edge disorder. Appl. Phys. Lett.

[25] Adam S, Cho S, Fuhrer MS, Sarma SD (2008). Density inhomogeneity driven percolation metal-insulator transition and dimensional crossover in graphene nanoribbons. Phys. Rev. Lett.

[26] Yao Z, Kane CL, Dekker C (2000). High‐field electrical transport in single‐wall carbon nanotubes. Phys. Rev. Lett.

[27] Chen Y‐F, Fuhrer MS (2005). Electric‐field‐dependent charge‐carrier velocity in semiconducting carbon nanotubes. Phys. Rev. Lett.

[28] Ferrari AC, Meyer JC, Scardaci V, Casiraghi C, Lazzeri M, Mauri F, Piscanec S, Jiang D, Novoselov KS, Roth S, Geim AK (2006). Raman spectrum of graphene and graphene layers. Phys. Rev. Lett.

[29] Fratini S, Guinea F (2008). Substrate‐limited electron dynamics in graphene. Phys. Rev. B.

[30] Fischetti MV, Neumayer DA, Cartier EA (2001). Effective electron mobility in si inversion layers in metal–oxide–semiconductor systems with a high-kappa insulator: The role of remote phonon scattering. J. Appl. Phys.

[31] Chen J‐H, Jang C, Xiao S, Ishigami M, Fuhrer MS (2008). Intrinsic and extrinsic performance limits of graphene devices on SiO_2_. Nat. Nanotechnol.

[32] Meric I, Han MY, Young AF, Özyilmaz B, Kim P, Shepard KL (2008). Current saturation in zero-bandgap, top-gated graphene field-effect transistors. Nat. Nanotechnol.

[33] Freitag M, Steiner M, Martin Y, Perebeinos V, Chen Z, Tsang JC, Avouris P (2009). Energy dissipation in graphene field-effect transistors. Nano Lett.

[34] Chae D-H, Krauss B, von Klitzing K, Smet JH (2010). Hot phonons in an electrically biased graphene constriction. Nano Lett.

[35] Berciaud S, Han MY, Mak KF, Brus LE, Kim P, Heinz TF (2010). Electron and optical phonon temperatures in electrically biased graphene. Phys. Rev. Lett.

[36] Balandin AA, Ghosh S, Bao W, Calizo I, Teweldebrhan D, Miao F, Lau CN (2008). Superior thermal conductivity of single-layer graphene. Nano Lett.

